# Femoral neck rotational osteotomy: a modified method for treating necrotic femoral heads with large and laterally located lesions

**DOI:** 10.1093/jhps/hnab016

**Published:** 2021-04-01

**Authors:** Junfeng Zhu, Kangming Chen, Jianping Peng, Yang Li, Chao Shen, Xiaodong Chen

**Affiliations:** 1 Department of Orthopaedics, Xinhua Hospital Affiliated to Shanghai Jiaotong University School of Medicine, Building 8, No.1665, Kongjiang Road, Shanghai 200092, China; 2 Department of Orthopaedics, Huashan Hospital, Fudan University, No. 12, Wulumuqizhong Road, Shanghai 200040, China

## Abstract

In this study, we retrospectively investigated the short-term outcome of femoral neck rotational osteotomy (FNRO) for treating necrotic femoral heads with large and laterally located lesions. Twelve necrotic femoral heads (ARCO stage II or III) with an average Kerboul angle of 210° underwent FNRO through surgical hip dislocation. By circumferential release of capsule and retinaculum, femoral neck osteotomy was performed at the base of femoral neck just 1.5 cm above lesser trochanter. The severed femoral neck was rotated with a mean angle of 120.4° and fixed with a mean varus angulation of 10.2°. Both Harris hip score and International hip outcome tool improved at a mean follow-up of 29 months. The average post-operative intact rate was 55.3%. Subsequent collapse or progression to osteoarthritis was found in four hips but only one hip failed with a Harris hip score of 44 and converted to hip replacement. Post-operative leg length discrepancy was 1.1 cm. Limp presented in seven hips. Six hips had osteophyte formation. FNRO through surgical hip dislocation had the advantages of safe exposure, direct visualization of necrotic lesion and high reorientation of healthy bone and articular cartilage on femoral head. We observed satisfactory short-term survivorship and improved patient-reported outcomes in necrotic femoral heads treated with FNRO.

## INTRODUCTION

Hip preservation surgery is preferred but remains a challenge for young patients with avascular necrosis of the femoral head (AVN). The survival rate of the femoral head decreases with increased size, advanced stage and lateralization of the necrotic lesion [1, 2]. Several osteotomies have been introduced for treating large lesions with type C according to Japanese investigation committee (JIC) classification [3]. Since the necrotic portion was moved away from the weight-bearing region, good results of transtrochanteric rotational osteotomy (TRO) and transtrochanteric curved varus osteotomy (TCVO) have been reported by Japanese surgeons after long-term follow-up. Two surgical techniques were emphasized for success of the osteotomy. First, the transposed intact area should be more than 36% of the weight-bearing region [4]. Second, the blood supply of proximal femur, especially the branches of the medial femoral circumflex artery (MFCA), must be carefully protected during osteotomy and transposition. Achieving these conflicting goals was a challenge for surgeons. Excessive rotation would tighten the retinaculum that contains the blood supply of proximal femur. Based on this point of view, an upper limit of 140° for posterior rotation of proximal femur was suggested by Sugioka in TRO procedure [5]. The retinaculum should be as tension-free as possible while sufficient viable bones were transposed into the weight-bearing zone.

Surgical hip dislocation is an approach that provides safe exposure and direct vision of hip joint [6, 7]. Theoretically, releasing the retinaculum could change the pathway of nutrient vessels to femoral head, which allows increased range of rotation to a sectioned femoral head. This has been confirmed in femoral head osteotomy, which was routinely performed in our department for treating Perthes disease and slipped capital femoral epiphysis. The branches of the medial femoral circumflex artery and inferior retinaculum were successfully protected during femoral head osteotomy and reduction of the slipped capital. Since 2016, we have attempted to treat type C (JIC) osteonecrosis with large lesions through this approach. The retinaculum was released during the surgery, and rotational osteotomy was performed at the base of the femoral neck. Few studies reported the results of treating AVN with femoral neck rotational osteotomy (FNRO) through surgical hip dislocation. Steppacher et al. [6] reported a promising short-term result of AVN treated through surgical hip dislocation in combination with subtrochanteric varus osteotomy in 10 of 13 hips. Regrettably, we failed to find a detailed description on how to release the retinaculum in their study. Hence, we retrospectively reviewed a series of AVN hips treated with FNRO in this study. We sought to answer the question as whether FNRO through surgical hip dislocation and release of the retinaculum results in: (i) enough viable bone transposed to the weight-bearing zone; (ii) improved patient-reported outcomes and less complications; (iii) progression to femoral head collapse or hip osteoarthritis.

## MATERIALS AND METHODS

### Patients

A consecutive series of 12 patients who underwent surgical hip dislocation with FNRO was retrospectively reviewed according to the ethical standards of Helsinki-Ethical Principles for Medical Research Involving Human Subject. All osteotomies were performed between October 2016 and December 2017 by a single senior surgeon (XD Chen). The inclusion criteria for the FNRO surgery were defined as followed: (i) a painful hip (visual analogue score > 4); (ii) age less than 55 years; (iii) ARCO stage II or III [8]; (iv) medium or large size of necrotic area with a Kerboul angle more than 160° [9]; (v) the location of necrotic area was classified as type C according to JIC classification. The exclusion criteria included hip dysplasia, Perthes disease and decreased joint space on conventional radiographs. Patient demographics including age, sex, body mass index, etiology, Kerboul angle, previous surgeries and follow-up period are shown in Table I.

**Table I:** Demographic data of the 12 patients

Parameters	Value
Age (years old)	29.6 ± 12.9 (17–51)^a^
Sex (male/female)	11/1
Body mass index (kg/m^2^)	22.7 ± 3.6 (16.6–27.8) *
Etiology (hips)	
Idiopathic	6
Steroids	3
Traumatic	2
Alcoholic	1
Kerboul angle (°)	210 ± 36 (181–300)^a^
Previous surgical procedures	
Internal fixation	2
Implant removal	1
Follow-up period (month)	29 ± 5.4 (24–40)^a^

^a^ Values are expressed as mean ± standard deviation with range in parentheses.

### Surgical technique

**Fig. 1.**
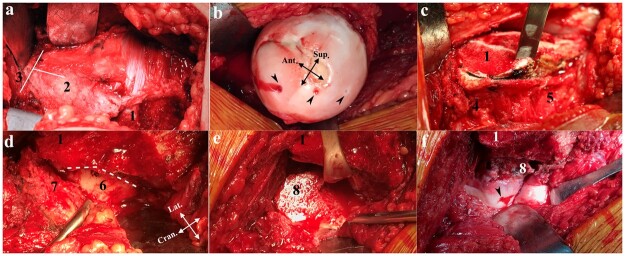
FNRO procedure in a right necrotic hip. (**a**) After a flat trochanteric osteotomy, anterior capsule was exposed with the right hip externally rotated. The white lines indicated a following proximal T-shaped capsulotomy. (**b**) After hip dislocation, the blood supply of femoral head was inspected by drilling holes on the femoral head with a 1.2-mm Kirschner wire. Arrow heads indicated the drilling holes. Normal bleeding was only showed in the anteroinferior quadrant. (**c**) After hip reduction, the soft tissues including muscles, capsules and periosteum were released as a whole from its distal attachment. (**d**) Circumferential release was performed from the level of less trochanter to the middle of femoral neck. Dashed lines indicated the following osteotomy site. (**e**) The femoral neck was severed at the base part and posteriorly rotated. (**f**) The severed femoral neck was fixed after a posterior rotation of 150°. Free bleeding was showed from a drilling hole on the new weight-bearing area of femoral head. 1 = trochanteric osteotomy; 2 = anterior capsule; 3 = acetabulum; 4 = attachment of piriformis and conjoint tendon; 5 = quadratus femoris; 6 = femoral neck; 7 = synovial sheath of the reflected portion of posterior capsule and the retinaculum; 8 = femoral neck osteotomy.

**Fig. 2.**
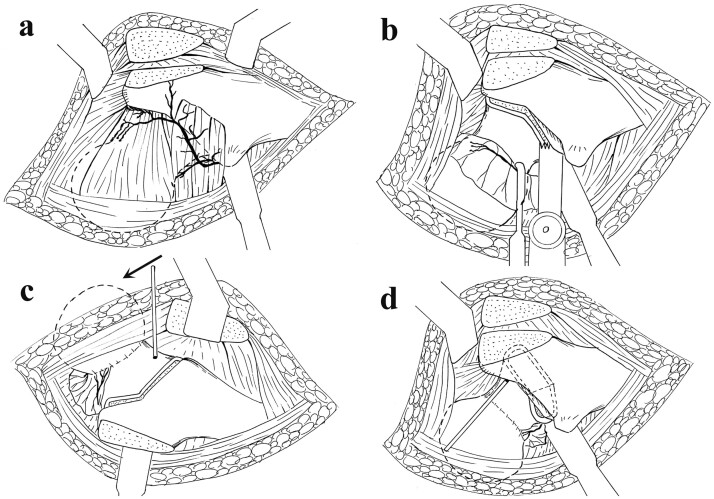
Diagrams showing FNRO in a right hip. (**a**) The nutrient vessels and osteotomized greater trochanter. (**b**) The released retinaculum and osteotomy line which goes in a way of an ice hockey stick. (**c, d**) Posterior rotation of the fragment.

**Fig. 3.**
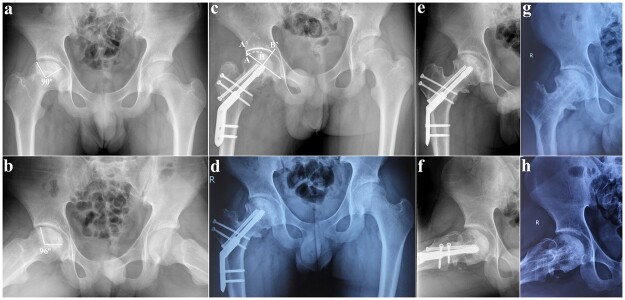
Radiographs of a necrotic femoral head treated with FNRO. Right hip pain presented in a 20-year-old man with a history of internal fixation for femoral neck fracture. (**a, b**) AP and frog-leg radiographs obtained 1.5 years after internal fixation showed femoral head necrosis on the right hip with a Kerboul angle of 186°. The ARCO stage was type III A. The JIC classification was type C2. (**c**) The immediate post-operative intact ratio was 69.1% by measuring AB/A′B ′ on an AP radiograph. (**d**) An AP radiograph obtained 1 year after FNRO. (**e, f**) AP and frog-leg radiographs obtained 2 years after FNRO. (**g, h**) AP and frog-leg radiographs obtained 3 years after FNRO. Osteophyte formation was observed however, neither subsequent collapse of the new weight-bearing area nor progression to osteoarthritis was found. At the last follow-up, the ARCO stage was type II and the JIC classification was type A.

A posterolateral approach and surgical dislocation was performed as previously reported [6]. The interval between the gluteus minimus and piriformis muscles was dissected after the tensor fascia lata was split. A flat trochanteric osteotomy was followed by a proximal T-shaped capsulotomy (Fig. 1a). After hip dislocation, the blood supply of femoral head was inspected by drilling holes on the femoral head with a 1.2-mm Kirschner wire (Fig. 1b). The size and location of the necrotic area was detected, which suggested the direction of the following rotation of severed femoral neck. Then the hip was reduced. The soft tissues including muscles, capsules and periosteum were released as a whole from its distal attachment (Fig. 1c). The circumferential release was performed from the level of lesser trochanter to the middle of femoral neck. The synovial sheath of the reflected portion of capsule and the retinaculum that contained the blood supply of femoral head were carefully released and protected (Figs 1d and 2). The FNRO was performed at the base of femoral neck just 1.5 cm above lesser trochanter. The osteotomy line consists of two parts and goes in a way of an ice hockey stick (Figs 1d and 2b). The first part was perpendicular to the axis of the femoral neck both in coronal and sagittal plan. The second part ran along the junction between the base of the femoral neck and greater trochanter. Then, the severed femoral head and neck was anteriorly or posteriorly rotated (Figs 1e and 2d) along the femoral neck axis with intentional varus angulation of 10–20° to move the necrotic portion of the femoral head from the weight-bearing region to a non-weight-bearing region. Free bleeding was identified from a drilling hole on the new weight-bearing area of femoral head (Fig. 1f). The FNRO was fixed with locking plate (Smith and Nephew, Memphis, TN) in two hips. A dynamic hip screw with a cannulated 6.5-mm screw (Synthes, West Chester, PA) was used in the other 10 hips (Fig. 3). The trochanteric osteotomy was fixed *in situ* with two 4.5-mm titanium alloy screws (Synthes, West Chester, USA).

### Rehabilitation

Quadriceps femoris exercise was encouraged after the surgery. Hip exercise and non-weight-bearing crutch walking started at four weeks post-operatively. Partial weight bearing was permitted three months post-operatively according to the bone healing at osteotomy site. Full weight bearing was permitted after six months.

### Follow-up

All hips were radiographically and clinically followed at three, six and twelve months post-operatively, and annually thereafter. Range of motion (ROM), Harris hip score (HHS) and International hip outcome tool (iHOT-33) were assessed pre-operatively and at the last follow-up. ROM referred to the sum of hip flexion-extension, internal and external rotation in 90° of flexion. AP pelvis, frog-leg radiographs and CT scans were acquired to assess the stage of osteonecrosis according to ARCO [8]. The ratio of intact area of femoral head in the weight-bearing area was post-operatively measured by using Sugioka’s method [10] (Fig. 3c). The varus angulation was calculated as the change of neck-shaft angle between pre-operative and post-operative radiographs. The subsequent collapse was identified when the maximum collapse of the transposed intact femoral head on weight-bearing region on AP radiograph was greater than 2 mm. Osteoarthritic progressions were diagnosed when the joint-space narrowing, increased osteophyte formation, subchondral bone cyst and femoral head asphericity were present. Failure was defined as a HHS score less than 70 or conversion to hip replacement. Leg length discrepancy (LLD) was evaluated as previously described [6].

### Statistical analysis

Statistical analyses were conducted using SPSS statistics 19 (IBM, Armonk, NY). Non-parametric statistical tests were qualified for this study because of the small sample size. The Wilcoxon test was used to compare ROM, LLD, HHS and iHOT-33 between the pre-operative status and the last follow-up. A *P* value < 0.05 was considered significant.

## RESULTS

The mean follow-up was 29 months. The results were shown in Table II. The average rotational angle was 120.4°. The mean varus angulation was 10.2° post-operatively. Bone union at the osteotomy site was achieved in all hips without additional neck-shaft angle change during follow-up. The intact articular surface was transposed to the weight-bearing area with an average intact rate of 55.3%. Then the immediate post-operative JIC classification was improved in 11 hips. LLD increased from 0.3 cm pre-operatively to 1.1 cm post-operatively. Limp presented in seven hips. Osteophyte formation presented in six hips. Subsequent collapse of the new weight-bearing area or progression to osteoarthritis in follow-up was found in four hips. One patient (a 32-year-old man) had severe pain from subsequent collapse of femoral head. The HHS score was 44 and iHOT-33 score was 21 at two years post-operatively. The hip failed and converted to total hip replacement. There were eight Type II hips, three Type IIIA hips and one Type IV hip at the last follow-up. We observed improvement of nine hips based on JIC classification including five hips with Type A, two hips with Type B and two hips with Type C1.

**Table II:** Clinical and radiographic evaluation pre-operatively and at the last follow-up

Parameters	Preoperative	Last follow-up	*P* value
Range of motion (°)	182.5 ± 12 (160–200)	152.9 ± 19.4 (95–170)	0.002
Harris hip score	55.7 ± 8 (43–69)	80 ± 12 (44–90)	0.003
International hip outcome tool	35.7 ± 11 (19–52)	69.3 ± 16.2 (21–86)	0.002
LLD (cm)	0.3 ± 0.3 (0–0.9)	1.1 ± 0.6 (0–2.2)	0.003
Rotation of femoral necks (°)		120.4 ± 29 (80–170)^a^	
°Anterior rotation in 4 hips		90 ± 8.2 (80–100)^a^	
°Posterior rotation in 8 hips		135.6 ± 22.3 (90–170)^a^	
°Varus angulation (°)		10.2 ± 8 (-5–30)^a^	
Rate of intact area (%)		55.3 ± 16.7 (34.3–85.7)^a^	
JIC location			
°A	0	5 {6^a^}	
°B	0	2 {4^a^}	
°C1	3	2 {1^a^}	
°C2	9	3 {1^a^}	
ARCO stage (hips)			
°II	3	8	
°III A	3	3	
°III B	6	0	
°IV	0	1	

Values in upper part were expressed as mean ± standard deviation with range in parentheses.

^a^ Values were evaluated immediately after the surgery.

At the last follow-up, the mean HHS score improved from 55.7 pre-operatively to 80 post-operatively, and the mean iHOT-33 score improved from 35.7 pre-operatively to 69.3 post-operatively. However, the average ROM decreased from 182.5° pre-operatively to 152.9° post-operatively.

## DISCUSSION

Osteotomies were suggested for treating AVN with Ficat stage of Type 2b or 3 [11, 12]. By releasing the retinaculum, FNRO through a surgical hip dislocation approach transposed enough viable bone to the weight-bearing zone of femoral head. Satisfactory short-term survivorship and improved patient-reported outcomes in this study suggested a promising new method of rotating (up to 170°) the severed femoral neck with considerable degrees without interrupting the blood perfusion of femoral head.

Morita et al. [13] investigated 111 hips which underwent TRO for treating AVN, the mean HHS significantly improved from 72 pre-operatively to 85 one year post-operatively. Hasegawa et al. [14] also reported an increased HHS from 64 pre-operatively to 84 at 5 years follow-up in 77 hips. The average pre- and post-operative HHS in this study was comparable to that in the previous studies. The post-operative iHOT-33 score in this study also significantly improved at the last follow-up. The less score of iHOT-33 indicated social, emotional and lifestyle concerns which prevailed in these young patients. The excellent long-term results of TRO reported by Japanese authors were not reproduced in European and North American studies [4]. Given the primary outcome of this study, FNRO have several potential advantages. Firstly, surgical hip dislocation allowed safe exposure and direct vision of hip joint. The blood supply to femoral head was hardly affected although the ligament teres was sacrificed [6, 15, 16], since the foveal artery was absent in adults or small in diameter when present [17, 18]. Dislocating femoral head did not influence the natural course and tension of the MFCA, as long as obturator externus was left attached [18, 19]. Necrotic area and healthy bone was directly visualized before osteotomy. The direction of rotation depended on the distance between intact healthy bone and acetabular roof, which was measured on radiographs pre-operatively and confirmed intra-operatively. Secondly, the tension of retinaculum and MFCA was reduced after releasing retinaculum during FNRO procedure. The deep branch of MFCA crosses posterior to the tendon of obturator externus and anterior to the superior gemellus, obturator internus and inferior gemellus [20]. Soft tissues including vessels, capsules and periosteum were carefully released from femoral attachment. Then the severed femoral neck could be intensively rotated without interrupting the blood perfusion of femoral head. In TRO, femoral head was rotated anteriorly by 60° to 90° [21], or posteriorly up to 140° [5]. In this study, however, the degree of rotation was 80° to 100° for anterior rotation, and 90° to 170° for posterior rotation. Thirdly, iatrogenic damage to the MFCA was not rare in intertrochanteric osteotomy [22, 23]. The deep branch of MFCA lies close to the trochanteric crest while the posterior aspect of femoral neck lacks retinacular vessels [19]. Therefore, the osteotomy site of this study was moved proximally from intertrochanter to the base of femoral neck after the released retinaculum was carefully protected. Finally, more intact femoral head (55.3%) in this study was moved to weight-bearing area, which helps to prevent subsequent collapse of the femoral head. Several studies suggested the transposed new intact area should exceed 34%, 36% or 40% of the weight-bearing region [10, 14]. In this study, 9 of 12 hips (75%) obtained an intact ratio of more than 40% post-operatively despite the mean Kerboul angle being 210°. This might be attributed to releasing the retinaculum.

Four hips in this study presented subsequent collapse of the new weight-bearing area or progressive osteoarthritis at the last follow-up, although only one of them failed. Type C2 was a risk factor for secondary collapse and failure [13, 22]. The failed hip might result from a larger Kerboul angle of 300° pre-operatively and a less intact ratio of 34.3% immediately after the surgery. A cut-off point of 41.9% was reported to prevent collapse and joint-space narrowing in 73 hips treated with TCVO [24]. However, neither TRO nor TCVO could obtain adequate weight-bearing area in necrotic hips with Type C2 location and large necrotic region. The average post-operative intact ratio was 55.3% in this study because intentional varus positioning of 10.2° was combined with retinaculum release and femoral neck rotation. At the last follow-up, JIC classification remained Type A or B in seven hips. Only one hip showed more advanced **ARCO** stage. Similar method was suggested in several studies as an important factor of success in TRO [25, 26]. Our result did not support Rijnen’s findings in which 15 of 24 hips failed although the post-operative intact ratio surpassed 33% [27].

Lee [28] reported a mean LLD of 0.83 cm in hips undergoing TRO. Post-operative increase of 1.1 cm in LLD was inevitable since combined varus fixation of femoral head fragment was performed in this study. Varus fixation of the proximal femur was associated with the risk of progressive varus deformity and delayed neck fracture. Neither of them was observed in our study. It might be explained by the strong fixation. However, the most important factor we believed was the proximal move of osteotomy site in this study which produced more distinct biomechanical advantage than TRO.

Limp was a frequent (58%) complication in this study. Steppacher et al. [6] reported a better result of 23% in 13 hips undergoing surgical dislocation and femoral varus osteotomy. Limp might be attributed to muscle weakness, increased LLD and decreased ROM. Late mobilization and inadequate physical therapy should be responsible for loss of ROM, especially the decreased hip flexion and internal rotation. Osteophyte formation in 6 of 12 hips was another reason. Additionally, the removed necrotic area might undermine the femoral head sphericity even if it has been rotated away from the weight-bearing region. The secondary collapse could be a potential risk for hip impingement, which also decreased the post-operative ROM. The concomitant femoroacetabular impingement was identified and treated in Steppacher’s series of AVN hips [6]. This might explain the better outcome in post-operative ROM and limp in their study. Finally, the function of ligament teres was sacrificed due to surgical hip dislocation. A deficient ligament teres might lead to hip instability, although ligament teres was not a primary restraint but rather a secondary stabilizer [29].

This study has several limitations. First, it included a small number of patients with a short-term follow-up. At the early stage of our practice, we were limited by both the learning curve and conservative patient selection. But our result was still promising since the symptomatic AVN hips mostly degraded within two years in the natural course [30]. Second, no control group of other preservative procedures could be matched in this study. So the outcome and survivorship of FNRO was directly compared with those in literatures. Third, the stage and location of the necrotic area were evaluated only by plain radiographs since MRI was not part of the clinical routine. The size of necrotic region might be underestimated both pre-operatively and post-operatively.

## CONCLUSION

Satisfactory short-term survivorship, improved HHS and iHOT-33 scores were identified in AVN hips treated with FNRO through surgical hip dislocation. With regard to the lateral location and large size of the necrosis lesions in this study, our result was promising although it did not achieve the superior outcome from femoral osteotomies in literatures. FNRO via a surgical hip dislocation approach had the advantages of safe exposure, protecting blood supply, direct visualization of necrotic lesion and high reorientation of the healthy bone on femoral head. Further investigation is warranted to determine the long-term outcome of FNRO.
